# Latin American registry of renal involvement in COVID-19 disease. The relevance of assessing proteinuria throughout the clinical course

**DOI:** 10.1371/journal.pone.0261764

**Published:** 2022-01-27

**Authors:** Raúl Lombardi, Alejandro Ferreiro, Daniela Ponce, Rolando Claure-Del Granado, Gustavo Aroca, Yanissa Venegas, Mariana Pereira, Jonathan Chavez-Iñiguez, Nelson Rojas, Ana Villa, Marcos Colombo, Cristina Carlino, Caio Guimarâes, Mauricio Younes-Ibrahim, Lilia Maria Rizo, Gisselle Guzmán, Carlos Varela, Guillermo Rosa-Diez, Diego Janiques, Roger Ayala, Galo Coronel, Eric Roessler, Serena Amor, Washington Osorio, Natalia Rivas, Benedito Pereira, Caroline de Azevedo, Adriana Flores, José Ubillo, Julieta Raño, Luis Yu, Emmanuel A. Burdmann, Luis Rodríguez, Gianny Galagarza-Gutiérrez, Jesús Curitomay-Cruz

**Affiliations:** 1 Department of Nephrology, Universidad de la República, Montevideo, Uruguay; 2 Clinical Hospital of Botucatu, School Medicine, HCFMB, University of Sao Paulo State UNESP, Brazil; 3 Hospital Obrero #2—C.N.S., School of Medicine, Universidad Mayor de San Simón, Cochabamba, Bolivia; 4 Universidad Simón Bolívar, Barranquilla, Colombia; 5 Hospital Nacional Arzobispo Loayza, Servicio de Nefrología, Lima, Perú; 6 School of Medicine, University of São Paulo, Brazil; 7 Division of Nephrology, Hospital Civil de Guadalajara, Guadalajara, Mexico; 8 Departamento de Nefrología, Hospital General de Agudos Dr Cosme Argerich, Buenos Aires, Argentina; 9 Serviço de Nefrología, Santa Casa de Jau, Jau, Brazil; 10 Department of Nephrology, Hospital Provincial, Rosario, Argentina; 11 Nefrologia, Pontificia Universidade Catolica do Rio de Janeiro, Rio de Janeiro, Brazil; 12 Internal Medicine, University of Rio de Janeiro, Rio de Janeiro, Brazil; 13 Nephrology Hospital Universitario Dr José Eleuterio González, Monterrey, Mexico; 14 Centros de Diagnóstico y Medicina Avanzada, Santo Domingo, República Dominicana; 15 Servicio de Nefrología, Hospital Italiano, Buenos Aires, Argentina; 16 Departamento de Medicina Interna, Salud Renal MSP, Asunción, Paraguay; 17 Department of Nephrology, Pontificia Universidad Católica de Chile, Santiago, Chile; 18 Servicio de Nefrología, Hospital Español, Montevideo, Uruguay; 19 Departamento de Nefrología, Hospital de Especialidades de las Fuerzas Armadas, Quito, Ecuador; 20 Servicio de Nefrología y Diálisis, Hospital Rojas, Buenos Aires, Argentina; 21 Nephrology, School of Medicine, University of São Paulo, Brazil; 22 Hospital Federal Cardozo Fontes, Universidade de Sá, Rio de Janeiro, Brazil; 23 Departamento de Nefrología, Hospital Regional General Dr Carlos Mac Gregor Sanchez Navarro, DF, Mexico; 24 Departamento de Nefrología, Hospital de Pediatría CMN Siglo XXI, DF, Mexico; 25 Department of Nephrology, School of Medicine, University of Sao Paulo, Sao Paulo, Brazil; 26 Centro Infantil del Riñón, Tucumán, Argentina; 27 Hospital Regional de Ica, Ica, Perú; 28 Hospital Nacional Hipólito Unanue, Lima, Perú; University of Florida, UNITED STATES

## Abstract

The Latin American Society of Nephrology and Hypertension conducted a prospective cohort, multinational registry of Latin American patients with kidney impairment associated to COVID-19 infection with the objective to describe the characteristics of acute kidney disease under these circumstances. The study was carried out through open invitation in order to describe the characteristics of the disease in the region. Eight-hundred and seventy patients from 12 countries were included. Median age was 63 years (54–74), most of patients were male (68.4%) and with diverse comorbidities (87.2%). Acute kidney injury (AKI) was hospital-acquired in 64.7% and non-oliguric in 59.9%. Multiorgan dysfunction syndrome (MODS) due to COVID-19 and volume depletion were the main factors contributing to AKI (59.2% and 35.7% respectively). Kidney replacement therapy was started in 46.2%. Non-recovery of renal function was observed in 65.3%. 71.5% of patients were admitted to ICU and 72.2% underwent mechanical ventilation. Proteinuria at admission was present in 62.4% of patients and proteinuria during hospital-stay occurred in 37.5%. Those patients with proteinuria at admission had higher burden of comorbidities, higher baseline sCr, and MODS was severe. On the other hand, patients with *de novo* proteinuria had lower incidence of comorbidities and near normal sCr at admission, but showed adverse course of disease. COVID-19 MODS was the main cause of AKI in both groups. All-cause mortality of the general population was 57.4%, and it was associated to age, sepsis as cause of AKI, severity of condition at admission, oliguria, mechanical ventilation, non-recovery of renal function, in-hospital complications and hospital stay. In conclusion, our study contributes to a better knowledge of this condition and highlights the relevance of the detection of proteinuria throughout the clinical course.

## Introduction

Coronavirus disease 2019 (COVID-19), caused by the coronavirus SARS-CoV-2 is an ongoing pandemic that entails high morbidity and mortality rates. Within a month after the first Latin American case was reported on February 2020 in Brazil, all countries in Latin America had reported cases of the novel COVID-19 [1]; and by June 2020 Latin America & The Caribbean became the worlds’ latest COVID-19 epicenter with the number of deaths in the region exceeding four million, or over 27% of the world’s covid-19 deaths [2]. Sadly, COVID-19 cases are still growing in the region due to constrained health systems, high prevalence of chronic conditions, delayed responses from governments, and widespread poverty and inequalities [3]. Growing evidence has demonstrated that kidney involvement, mainly acute kidney injury (AKI) is prevalent among patients with COVID-19, particularly among critically ill patients affecting approximately 20–40% of patients admitted to intensive care units [4, 5]. Similar to the association of AKI with other forms of community-acquired pneumonia [6], AKI is now recognized as a common complication of COVID-19 and as with AKI from other causes, is associated with adverse outcomes [7]. On the other hand, disturbances of the urinary sediment has not been extensively studied in spite its known value. Available information on epidemiology and risk factors for AKI in the region is generally scarce, and this situation has not improved during the COVID-19 pandemic [8]. Knowledge of patient characteristics, risk factors and adverse outcomes, as well as regional peculiarities is key in the fight against this new disease. Accordingly, the AKI Committee of the Latin American Society of Nephrology and Hypertension (SLANH) carried out a prospective cohort study with the aim to describe clinical characteristics, adverse outcomes and its associated risk factors in patients with kidney impairment associated to Covid-19

## Methods and patients

This is an observational, prospective, longitudinal, multinational cohort study based on a registry carried out between May 1^st^ 2020 and December 31th 2020. An open invitation to participate in the Registry was made through the SLANH website, the National Societies of Nephrology and by personal email sent to members of *RedIRA* (Latin American AKI-Network) a networked learning tool of SLANH (http://redira.slanh.net/). Participation in the Registry was voluntary, without any incentive or economic benefit for patients or investigators. Data were obtained from the clinical record of patients and were entered online by the participants in a Surveymonkey® platform specifically designed for this purpose (https://es.surveymonkey.com/r/L6PVMGQ). The form has six sections that include: 1) country and city of residence, plus demographic data; 2) comorbidities and condition at admission; 3) laboratory at admission; 4) characteristics and causes of AKI; 5) ICU admission, mechanical ventilation (MV) and in-hospital complications; 6) condition at discharge (**S1 Table**). Potential bias could exists considering that is a registry of non-consecutive patients. Periodical reminders were sent to participants in order to reduce the rate of non-response and loss of follow-up. We also sent follow-up reminders of incomplete cases throughout the registry with the intention of completing missing cases. Inclusion criteria included adult and children with COVID-19 infection confirmed by RT-PCR of nasopharyngeal swabs and acute kidney injury (AKI) defined by KDIGO 2012 sCr criteria, and/or urinary sediment abnormalities (proteinuria/hematuria). Exclusion criteria: included patients with chronic kidney disease (CKD) stage 5, patients on chronic dialysis or transplanted.

### Definitions

Acute kidney injury was identified according to KDIGO definition when occurred an increase in serum creatinine level ≥ 0.3 mg/dL within 48 hours or by 50% within 7 days and staged in the three KDIGO categories. For the detection of proteinuria or hematuria, the semiquantitative dipstick test was used; data were requested at admission in all patients and during hospital stay in those patients without proteinuria at admission. Renal recovery was considered when sCr returned to baseline or reference value or lower. Non-recovery was established when sCr did not decrease or if the patient remained on dialysis at hospital discharge. AKI was considered as community-acquired (CA-AKI) when patient had an elevated sCr at admission or within the first 24 hours of admission and hospital-acquired (HA-AKI) when AKI developed during hospital stay. Condition on admission to the hospital was classified in three categories: *mild*, if the patient was admitted to a conventional ward without need for oxygen therapy; *moderate*, if the patient required oxygen therapy; and *severe*, if the patient was admitted to the intensive care unit (ICU).

Bioethical considerations: the Institutional Review Board of the *Clínica Los Olivos*, Cochabamba, Bolivia (contact Dr. Esdenka Vega, administracion@clinicalosolivos.com) approved the study. The informed consent was considered not mandatory by the reference IRB given the observational characteristic of the study. Protocol and forms are available on the study’s website (https://slanh.net/registro-latinoamericano-ira-covid-19/). Confidentiality of information was appropriately protected by de-identification of data. No personal information of patients was included in the form, so that the data were completely anonymized when it was entered into the database. Together with the registry, a short survey aimed to know working conditions, difficulties and challenges faced in the course of the pandemic was carried out during the study (**S2 Table**).

### Statistical analysis

Quantitative variables are presented as median and interquartile range (IQR) according to their distribution, and the categorical variables as number and proportions. Kolmogorov-Smirnov test was used to explore the data distribution. For the bivariate comparisons between groups, the Chi square test was used for categorical variables and the *U* Mann-Whitney test or the Kruskal-Wallis test for quantitative variables. Odds ratio and 95% Confidence Interval (CI) were also calculated. Statistical tests were two-sided and significance was considered with a probability of null hypothesis ≤5%. A multiple logistic regression model was performed to predict mortality using the forward conditional elimination method. The probability for entering the model was set at 0.10 and for elimination at 0.20. Variables that reached a significance *<*0.10 in the univariate analysis were included. The statistical package IBM SPSS Statistics Base version 22 NY, USA, was used for data processing and for statistical analysis.

## Results

The overall results of the entire population are presented all together and some subgroups considered of particular interest are described separately.

### Descriptive analysis of the entire population

**Fig 1:**
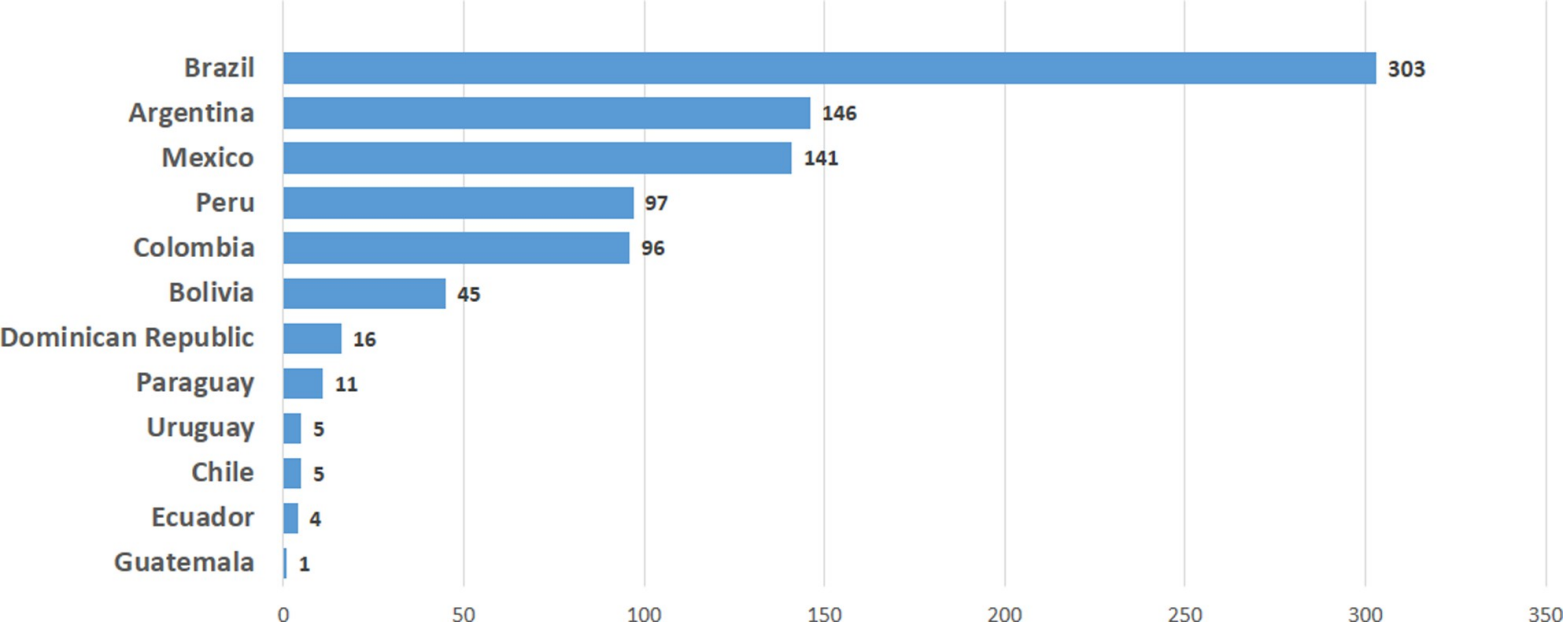
Patients per country.

Forty participants from 52 cities in 12 countries entered 967 patients in the Registry. Ninety-seven patients were excluded due to lack of data on outcomes, remaining 870 patients for the analysis. Median age was 63 (54–74) years; 595 were male (68.4%). Distribution by country of enrolled patients is shown in **Fig 1.** Patients’ general characteristics are shown in Table 1.

**Table 1:** Baseline data, outcome and missing data for variables of general population.

	All (870)	Alive (326)	Dead (544)	*P*	Missing data
**Age years, median (IQR)**	63 (54–74)	59 (48.4–69)	65 (57–75)	<0.001	63
**Male sex n (%)**	541 (62.5)	212 (65.2)	383 (70.8)	NS	4
**Comorbidities n (%)**					0
**Hypertension**	536 (61.1)	191 (58.6)	345 (63.4)	NS	
**Diabetes**	341 (39.4)	118 (36.2)	225 (41.4)	NS	
**Obesity**	278 (32.0)	79 (24.2)	199 (36.6)	<0.001	
**Cardiovascular disease**	135 (15.5)	50 (15.3)	67 (12.3)	0.038	
**Chronic Kidney Disease**	117 (13.4)	41 (12.6)	94 (17.3)	NS	
**COPD**	60 (6.9)	20 (6.19	40 (7.4)	NS	
**Immunodepression**	23 (2.7)	11 (3.4)	13 (2.4)	NS	
**None**	111 (12.8)	48 (14.7)	63 (11.6)	NS	
**Etiological risk factors n (%)**					0
**SARS-CoV-2 MODS**	515 (59.2)	139 (42.6)	376 (69.1)	<0.001	
**Hypovolemia/dehydration**	311 (35.7)	55 (47.5)	156 (28.7)	<0.001	
**Sepsis MODS**	254 (29.2)	47 (14.4)	207 (38.1)	<0.001	
**Nephrotoxic drugs**	182 (20.9)	83 (25.5)	99 (18.2)	0.007	
**Rhabdomyolysis**	10 (1.2)	4 (1.2)	6 (1.1)	NS	
**Time Covid-19 to admission, days**	2 (0–4)	1.5 (0–5)	2 (0–4)	NS	122
**Time Covid-19 to AKI onset, days**	3 (1–7)	2 (1–5)	4 (1–8)	<0.001	104
**Condition at admission, n (%)**					2
**Mild**	121 (13.9)	85 (26.2)	36 (6.6)	<0.001	
**Moderate**	384 (44.2)	147 (45.2)	237 (43.6)	NS	
**Severe**	363 (41.8)	93 (28.6)	270 (49.7)	<0.001	
**Laboratory at admission**					
**sCr mg/dL**	1.20 (0.90–1.93)	1.32 (0.97–2.31)	1.19 (0.90–1.80)	<0.001	5
**Serum potassium mmol/L**	4.2 (3.9–4.8)	4.2 (3.9–4.8)	4.2 (3.9–4.8)	NS	66
**WBC mm3**	10400 (7800–14137)	9550 (6975–12710	11200 (8225-	<0.001	12
**Platelets x 1000**	236 (174–302)	233 (174–294)	15795)	NS	6
**INR**	1.1 (1.00–1.26)	1.1 (1.0–1.2)	239 (174–307)	NS	294
**Ferritine (ng/ml)**	1067 (554–1895)	840 (366–1307)	1.1 (1.02–1.29)	<0.001	398
**pH**	7.39 (7.30–7.43)	7.38 (7.30–7.44)	1285 (689–2000)	NS	161
**HCO3 mmol/L**	22 (19–24)	21 (18.5–24)	7.39 (7.30–7.42)	NS	164
**AST (U/L)**	38 (23–63)	35.5 (25–61)	22 (19.24)	NS	143
**ALT (U/L)**	32 (20–55)	32 (20–52)	39 (23–64)	NS	145
**CK (U/L)**	168 (81–421)	123 (60–250)	32 (20–60)	0.004	549
**Proteinuria at admission, n (%)**	245 (62.3)	74 (50.0)	171 (69.8)	<0.001	482
**Hematuria at admission, n (%)**	144 (36.9)	46 (31.3)	98 (68.0)	NS	480
**sCr peak (mg/dL)**	3.60 (1.97–5.20)	2–15 (1.49–4.40)	4.10 (2.80–5.47)	0.003	7
**Last available sCr (mg/dL)**	2.40 (1.10–4.10)	1.08 (0.81–1.95)	3.40 (2.11–4.90)	<0.001	27
**Lowest PaO**_**2**_ **/FiO**_**2**_	134 (112–180)	164 (123–237)	123 (108–158)	<0.001	325
**Lowest PEEP (cmH**_**2**_ **0)**	14 (12–15)	13 (11.5–15)	14 (2–15)	NS	369
**Proteinuria *de novo*, n (%)**	39 (37.5)	16 (25.8)	23 (54.8)	0.015	766
**Hospital-acquired AKI, n/%**	547 (64.7)	148 (48.2)	399 (74.0)	<0.001	24
**Non-oliguric AKI, n (%)**	521 (60.8)	244 (76.0)	277 (51.7)	<0.001	13
**Kidney replacement therapy, n (%)**	402 (46.5)	87 (27.0)	315 (58.0)	<0.001	5
**AKI recovery, n/%**	289 (35.2)	229 (76.3)	60 (11.5)	<0.001	48
**ICU, n/%**	622 (73.8)	138 (42.7)	484 (93.1)	<0.001	27
**Mechanical ventilation, n (%)**	628 (74.1)	136 (42.5)	492 (93.2)	<0.001	22
**In-hospital complications, n (%)**					
**Sepsis**	439 (50.5)	79 (25.1)	360 (67.2)	<0.001	19
**Infection**	76 (8.7)	54 (17.4)	22 (4.3)	<0.001	44
**Venous thrombosis**	47 (5.4)	13 (4.2)	34 (16.6)	NS	41
**Other**	145 (16.7)	51 (16.5)	94 (18.1)	NS	41
**No complications**	161 (18.5)	110 (34.9)	51 (9.9)	<0.001	39
**Length-of-hospital stay (days)**	13 (8–23)	14 (8–28)	13 (8–21)	0.046	NS

Values are presented as n (proportion) or median (interquartile range). COPD = chronic obstructive pulmonary disease; MODS = multiorgan dysfunction syndrome; sCr = serum creatinine; WBC = white blood count; INR = international normalized ratio; AST = aspartate aminotransferase; ALT = alanine transaminase; CK = creatine kinase; PEEP = positive end-expiratory pressure; PaO2/FiO2 = arterial/inspired O_2_ ratio; ICU = intensive care unit, nephrotoxic drugs = NSAID, ACEI, vancomycin, aminoglycosides, antiviral agents.

Of the 870 patients 759 (87.2%) had one or more comorbidities being hypertension, diabetes and obesity were the three most common. Time between diagnosis of COVID-19 and hospital admission was 2 (0–4) days and condition at hospital admission was mild in 121 (14.0%); moderate in 384 (44.2%) and severe in 363 (41.8%).

Of note, about half of patients had a serum creatinine at admission within normal values. In 393 patients a urine dipstick test was performed, in 245 (62.0%) of them it was detected some degree of proteinuria: + in 151; ++ in 64; +++ in 22; and ++++ in 5 patients. Hematuria was present in 144 out of 390 cases (36.9%). Thirty-nine out of 104 evaluable patients (37.5%) developed proteinuria during their hospital stay. AKI was hospital-acquired in 547 (62.9%). Median time between diagnosis of COVID-19 and the onset of AKI was 3 (1–7) days. As expected, etiology of AKI was multifactorial or was atributed to more than one exposure in most of the cases. The most common cause of AKI was multi-organ dysfunction (MODS) attributable to SARS-CoV-2 infection. In the majority of cases, AKI was non-oliguric (59.9%) and hospital acquired (64.7%). The proportion of KDIGO stage 1, 2 and 3 were 25.8%, 14.5%, and 59.7% respectively. Four hundred two patients (46.2%) required acute kidney replacement therapy (KRT). Need of acute KRT was associated to higher level of admissions’ sCr (OR 1.32, 95% CI 1.00–1.74, p = 0.049); lower level of admission pH (OR 0.01, 95% CI 0.00–0.44, p = 0.016); hospital-acquired AKI (OR 4.26, 95% CI 1.90–9.56, p = 0.000); COVID-19 MODS (OR 5.52 95%CI 2.82–10.82, p = 0.000); and oliguria (OR 5.97, 95% CI 2.89–12.34, p = 0.000). Forty-three patients who had indication for KRT didn’t receive it (4.9%); this group of patients had higher mortality than the general population (86% vs 62.5%, *P* = 0.010). The reason for withholding KRT was not recorded in the form but presumably was due to shortage of resources, as can be found in the companion survey answered by 140 Latin American nephrologists. Twenty-six nephrologists (18.3%) raise the issue that they weren’t able to provide KRT due to lack of resources (**S2 Table**). The most common type of KRT was intermittent hemodialysis (IHD) (32.4%), followed by prolonged intermittent kidney replacement therapy (PIKRT) (15.2%), continuous kidney replacement therapy (CKRT) (7.2%) and peritoneal dialysis (PD) (1.2%). In the remaining 0.4% cases other procedures such as hemofiltration and hemoadsorption was performed. Patients were treated for SARS-CoV-2 infection with steroids in 73.9%; oseltamivir in 18.7%; chloroquine-hydroxychloroquine in 12.5%; ivermectine in 6.8%; tocilizumab in 1.5%; remdesivir and linipovir-ritonavir in 0.6%. It should be highlighted that in a large proportion of patients the option “other treatment” was selected in 46.9% with no further clarification. Most of the patients had complications during the hospitalization prevailing sepsis in 50.4% of cases followed by infection without sepsis (8.7%) and deep venous thrombosis (5.4%). Clinical course was severe as expressed by the high number of critically ill patients needing mechanical ventilation (71.5% and 72.2% respectively). It should be noted that 6 patients were placed on mechanical ventilation outside the ICU. Renal recovery was observed in 35.2% of patients. Characteristics of patients with and without renal recovery are shown in detail in Supplementary material (**S3 Table**). All-cause in-hospital mortality was 62.5% (544 out of 870 patients). Of the 544 deceased patients 479 (88.0%) died in ICU. Among survivors, 135 (41.4%) were in ICU and could be discharged alive from the hospital. Table 2 shows the variables associated to mortality in the logistic regression model. Variables with more than 30% missing data or inconsistency were excluded from the model (ferritine, platelets count, INR, CK, PaO_2_/FiO_2_).

**Table 2:** Risk factors independently associated to in-hospital mortality. Multivariable logistic regression analysis. Variables entered in the model are those showed in Table 1.

	OR (CI 95%)	p
Age (yrs)	0.975 (0.955–0.996)	0.017
Sepsis-MODS as cause of AKI	3.367 (1.689–6.7129)	0.001
Severe condition at admission	3.697 (1.692–8.077)	0.001
Oliguric AKI	2.045 (1.094–3.821)	0.025
Non recovery of renal function	21.970 (12.195–39.578)	<0.001
Mechanical ventilation	15.790 (7.285–34.225)	<0.001
In-hospital complication	2.375 (1.052–5.359	0.037
Length of hospital stay (days)*	1.042 (1.023–1.061)	<0.001

For each day of hospital stay; MODS: multiorgan dysfunction syndrome.

### Subgroup I. Patients with proteinuria at admission

Characteristics of subgroup I are shown in Table 3.

**Table 3:** Clinical characteristics of patients with proteinuria at admission. Subgroup I.

Variable	Proteinuria	No proteinuria	p
	245	148	
Comorbidities n (%)			
Hypertension	165 (67.3)	85 (57.4)	0.039
Chronic kidney disease	54 (22.0)	7 (4.7)	<0.001
No comorbidities	23 (9.4)	24 16.2)	0.032
Cause of acute kidney injury n (%)			
SARS-CoV-2 MODS	162 (66.1)	83 (56.1)	0.030
sCr at admission mg/dl	1.39 (0.90–2.64)	0.99 (0.80–1.30)	<0.001
Serum potassium at admission	4.35 (4.00–5.02)	4.10 (3.80–4.40)	<0.001
sCr peak mg/dL	4.10 (2.70–5.55)	3.00 (1.50–4.90)	<0.001
Kidney replacement therapy n (%)	136 (56.0)	60 (40.8)	0.006
Renal function recovery n (%)	59 (25.5)	61 (47.7)	<0.001
ICU admission n (%)	195 (79.9)	100 (67.6)	0.02
Mechanical ventilation	198 (81.1)	101 (68.2)	0.013
Vasopressors n (%)	158(67.2)	74 (53.2)	0.005
In-hospital complication n (%) Infection	23 (9.4)	33 (22.7)	0.001
Last available Scr mg/dL	3.00 (1.60–4.50)	1.56 (0.90–3.14)	<0.001
Mortality n (%)	171 (69.8)	74 (50.0)	<0.001

A regression model including variables that reach statistical significance in the univariate analysis with mortality as outcome is shown in Table 4.

**Table 4:** Variables associated independently with mortality. Patients with proteinuria at admission. Variables entered in the model are listed in S4 Table.

Variable	OR (95% CI)	*P*
Age yrs	0.943 (0.916–0.972)*	<0.001
Sepsis MODS	4.156 (1.274–13.558)	0.018
Kidney replacement therapy	2.850 (1.150–7.062)	0.024
Mechanical ventilation	18.600 (5.236–66.076)	<0.001
Non recovery of renal function	51.009 (19.456–134.209)	<0.001

*For each year of age. MODS = multiorgan failure.

In summary, this subgroup of patients had higher burden of comorbidities, and elevated sCr and potassium at admission. AKI was linked to COVID-19 MODS and showed a severe course of the disease. The greatest mortality risk was related to severity of organ dysfunction and kidney injury. Within the Subgroup I, 34 out of 393 patients did not developed AKI. As expected, those cases had milder forms of COVID-19 at admission and had a less severe clinical course during hospitalization (need of ICU, MV, vasopressors, and mortality).

### Subgroup II. Patients with normal urinary sediment on admission and *de novo* proteinuria during hospital stay

Of 104 patients in whom urinary sediment was assessed during their hospitalization, 39 (37.5%) of them developed *de novo* proteinuria. Clinical characteristics are shown in Table 5.

**Table 5:** Characteristics of patients who developed *de novo* proteinuria. Subgroup II.

Variable	Proteinuria	No proteinuria	P
	39	65	
Male n (%)	33 (84.6)	43 (66.2)	0.032
Comorbidities n (%)			
Hypertension	27 (69.2)	30(46.2)	0.018
No comorbidities	3 (7.7)	16 (24.6)	0.025
Cause of acute kidney injury n (%)			
Dehydration/volume depletion	5 (12.8)	30 (46.2)	<0.001
SARS-CoV-2 MODS	37 (94.9)	26 (40.0)	<0.001
Nephrotoxic drugs	2 (5.1)	14 (21.5)	0.020
sCr at admission mg/dL	0.90 (0.90–1.00)	1.00 (0.80–1.40)	0.016
sCr peak mg/dL	4.80 (3.40–6.20)	1.80 (1.05–3.70)	<0.001
Kidney replacement therapy n (%)	33 (84.6)	14 (21.5)	<0.001
Renal function recovery n (%)	15 (31.3)	33 (50.8)	0.022
ICU admission n (%)	37 (94.9)	23 (35.4)	0.000
Mechanical ventilation n (%)	37 (94.9)	26 (40.0)	0.000
Vasopressors n (%)	31 (81.6)	17 (26.1)	0.000
In-hospital complications n (%)			
Sepsis	19 (48.7)	16 (25.0)	0.013
Infection	3 (7.7)	21 (32.3)	0.002
No complications	2 (5.1)	17 (26.1)	0.004
Last available sCr mg/dL	2.90 (1.10–3.70)	1.00 (0.80–1.65)	0.000
Hospital lenght-of-stay days	22 (14–37)	10 (8–17)	0.000
Mortality n (%)	23 (59.0)	19 (29.2)	0.003

MODS = multiorgan dysfuntion síndrome; ICU = intesive care unit; sCr = serum creatinine.

In brief, prevalence was higher in males, the sCr at admission was normal and the predominant cause of AKI was MODS associated to COVID-19. The clinical course was characterized by higher KRT requirement, higher incidence of organ dysfunction and complications, less recovery of renal function and higher mortality. In the multivariable adjusted logistic analysis with mortality as outcome remained in the model age, oliguria and need of mechanical ventilation (Table 6).

**Table 6:** Variables associated independently with mortality. Variables entered in the model are listed in S5 Table.

	OR (95% CI)	*P*
Age yrs	0.946 (0.905–0.987)	0.011
Oliguric AKI	4.398 (1.128–17.138)	0.003
Mechanical ventilation	15.243 (1.638–141.837)	0.017

### Critically ill patients

A large proportion of patients needed ICU admission (622, 71.5%) and 628 (72.2%) were placed on mechanical ventilation. **S6 Table** shows the different characteristics between critically ill and no critically ill patients. Obesity, proteinuria, hospital acquired AKI and MODS related AKI, delay in hospital admission, higher values in white blood cells and ferritine and, as expected, higher mortality were more common in critically ill patients.

## Discussion

In early December 2019, an outbreak of an acute respiratory infection caused by SARS-CoV-2 virus occurred in Wuhan City, China, which rapidly spread to other regions of China and later globally constituting a pandemic as was declared by WHO on March 11, 2020. The respiratory system is the primary target of the virus, but it was increasingly recognized that it could affect other organs, including kidneys leading to proteinuria, hematuria and AKI [9–11]. On the other hand, kidney impairment and mortality increases with disease severity [5, 12, 13]

The real incidence of AKI in COVID-19 is cause of debate. Early studies from China showed low AKI incidence (0.5%) in a series of 1,099 hospitalized patients [14]. On the other hand, a large series of 5,449 patients admitted to 13 academic and community hospitals in New York, identified 1,993 patients with AKI (36.6%) [5], an incidence close to the 33.9% reported in 35,302 patients from the largest hospital discharge database in USA (Premier Healthcare Database) [15].

Currently, the Americas became the epicenter of COVID-19 pandemic being the impact devastating on the Latin America subcontinent [16–18]. The rapid spread of the infection, the work overload of health teams, the uneven level of healthcare coverage and health systems in the region, and the differences in strategies in the fight against the pandemic between countries; partly explains the lack of data in a region, that otherwise has scarce general epidemiological information on AKI [19].

In order to improve gaps in the knowledge of kidney disorders associated with COVID-19 in the region the AKI Committee of the SLANH conducted a Registry that gathered data of 870 patients from 57 cities distributed in 12 countries in which the elderly male prevailed. Our population showed a high burden of comorbidities, like 87.2% had at least one associated disease, mainly hypertension, diabetes and obesity. Moreover, severe clinical condition at hospital admission was also predominant (86.0%). Finally, time elapsed between diagnosis of COVID-19 and hospitalization was short, which can be read as a marker of severity of the disease. It is not surprising therefore, that in-hospital mortality was high (62.5%), greater than reported in other studies [5, 12]. In contrast with the severity of SASR-CoV-2 infection at admission, renal and metabolic condition appeared to be less affected. In most of the cases, sCr level was near normal, as well as serum potassium and acid-base status. However, evidence of kidney impairment at admission was demonstrated since in 62.4% of the patients in whom a urine dipstick test was performed had proteinuria. With regard to AKI profile, the onset occurred shortly after COVID-19 diagnosis and was linked in the vast majority of cases to COVID-19 MODS and hypovolemia. As previously reported, the onset of AKI was mainly during hospital stay with a prevailing a non-oliguric and severe pattern [5]. An additional burden of seriousness was due to in-hospital complications, mostly sepsis (50.4%). Of note, 43 patients needing KRT didn’t received probably due to limited availability of resources in some centers. Similarly, some patients who required ICU and/or mechanical ventilation were not admitted or placed on mechanical ventilation due probably to the same reason. In the same line of reasoning, could be explained the reason why 6 patients received mechanical ventilation outside ICU.

In previous studies variable degrees of proteinuria were reported among COVID-19 patients, ranging between 33% and 74% with a higher incidence among AKI stages 2–3 [11] and it was invariably associated to worse outcome [5, 9]. Histopathological studies showed a diversity of presentations like collapsing glomerulopathy, thrombotic microangiopathy, minimal change disease, immune-mediated glomerulopathy and acute tubular necrosis [20, 21]. We assessed the presence of proteinuria at admission and/or through patients’ hospital stay (*de novo* proteinuria). By doing that, we identified two patterns of patients with different characteristics between them. The first group of patients, which had proteinuria at admission, had more comorbidities, were admitted with impaired renal function, had a worse course of their illness and had higher mortality rate as shown in Table 5. In spite in the multivariate analysis the presence of proteinuria was not associated independently to mortality, it certainly should be read as a surrogate marker of severity. On the other hand, the second group of patients with *de novo* proteinuria, had lower burden of comorbidities and normal kidney function at hospital admission, which could have predicted a better outcome. However, in most of the cases their clinical condition worsened requiring multi-organ support. They also showed prolonged hospital stay, recovered renal function less frequently and had a higher mortality rate (Table 6). Even though proteinuria *de novo* didn’t remain in the regression model, it should be considered also a marker of disease severity. To our knowledge, the latter pattern of proteinuria which is associate with poor outcomes, has not been reported previously and is a relevant finding of our study. Although the low number of cases limits the conclusions, it should be pointed out that this group of patients had worse outcomes (severity of AKI and Covid-19) in spite having more favorable conditions at admission (less comorbidities and lower sCr)In both groups of proteinuria, SARS-CoV-2 MODS was recognized by far as the main cause of AKI that strongly suggests glomerular and tubular damage due to severe inflammation, together with associated hemodynamic disturbances. These results lead us to strongly recommend including proteinuria assessing at admission and during hospital stayin the routine evaluation of COVID-19 patients for a timely detection of kidney injury. In fact and taking into account the potential role of proteinuria de novo as an early marker of kidney injury, the SLANH-AKI Commission launched an ongoing prospective multicenter multinational study looking at development of new proteinuria in hospitalized Covid-19 patients in order to confirm or not our findings.

As previously reported, AKI is associated to higher risk of death in COVID-19 patients [5, 15, 22]. Our study found higher than usual mortality rate, probably due to a selection bias since our Registry is an open repository of patients provided by selected physicians (nephrologists). We found as many as 24 variables associated with mortality in the univariate analysis. Unfortunately, some of them as proteinuria and ferritine could not be included in a regression model because of inconsistence of data or high number of patients with missing data. With these limitations, we identified eight variables that were independently associated to in-hospital mortality. Those are: the patient’s age (older age, OR 0.975); etiology of AKI (septic MODS, OR: 3.367); severity of the disease (condition at admission, OR: 3.697; mechanical ventilation, OR: 15.790; length-of-hospital stay, OR: 1.042; in-hospital complications, OR: 2.375); and severity of AKI (oliguria OR: 2.045; non-recovery of renal function, OR: 21.970; the latter being the most ominous predictor of death). Of note, data regarding sCr requested at three time points (admission, peak, discharge) did not enter in the model.

Our study has some limitations. First, although the design of the study as a repository promoted the individual participation, on the other hand entailed some heterogeneity in the quality of information with some variables having high number of missing data which forced us to exclude them from the analysis. Therefore, potential unmeasured confounder may not have been identified. Second, patients’ information was provided by the participants and not as a result of the review of clinical charts by the research team. Third, the limited ability to access the universe of millions patients did not allow us to establish incidence, which is a very relevant information, particularly considering that AKI incidence could be as low as 0.5% and as high as 36.6%. We also acknowledge some strengths. First, to date this is the largest multicenter study involving a large number of countries and cities from Latin America. Second, data requested were organized in six forms with 47 questions with the aim to fully explore the more relevant problems related to SARS-CoV-2 infection and kidney disease. With this approach, two different patterns of patients with proteinuria having relevant prognostic value were clearly identified.

In conclusion, this is the first multinational Registry of patients with renal impairment associated to SARS-CoV-2 infection in Latin America. The data of 870 patients from 12 countries generally shows similar disease profile to previously reported series regarding to demographic, underlying condition, AKI features, impact on distant organs and mortality. The study highlights the value of assessing proteinuria throughout the clinical course for a timely identification of renal impairment and potential adverse outcomes. Our results cannot be generalized, as it is a repository of patients that partially represents the universe of this condition. However, this study demonstrates the feasibility of carrying out similar studies in order to expand the knowledge of this new disease that remains to be fully characterized.

## Supporting information

S1 Dataset Database. (SAV)

S1 Table Form for data collection. (DOCX)

S2 Table Results of an open survey of Latin-American nephrologist participants and no participants of the Registry coming from 14 countries. Answers are expressed in percentage.
(DOCX)

S3 Table Features of patients with recovered and non-recovered renal function. (DOCX)

S4 Table Clinical characteristics of critically ill patients. (DOCX)

S5 Table Risk factors for mortality in patients assessed for proteinuria during hospital stay. Variables entered in the regression logistic model.
(DOCX)

S6 Table Clinical characteristics of critically ill patients. (DOCX)
