# Supplementary material for: Latin American registry of renal involvement in COVID-19 disease. The relevance of assessing proteinuria throughout the clinical course
Source: PLoS One. 2022 Jan 27;17(1):e0261764. doi: 10.1371/journal.pone.0261764 (PMC8794101; doi:10.1371/journal.pone.0261764)
Supplement: S1 Table — (DOCX) [file pone.0261764.s002.docx]

# **S1 Table. Form for data collection**

*1. Demographic data and location*

a. Age

b. Sex

c. City / town of residence

d. Country

*2. Comorbidities and condition at admission*

a. Comorbidities (hypertension, diabetes, obesity, chronic heart failure, chronic kidney disease, chronic obstructive pulmonary disease, immunodepression)

b. Days between COVID-19 diagnosis and admission to hospital

c. Hospital admission date

d. Condition at admission (mild, moderate severe)

*3. Laboratory at admission*

i. Serum creatinine

ii. Potasemia

iii. Proteinura at admission and *de novo* during hospital stay

iv. Hematuria

v. White blood cells count

vi. Lymphocytes count

vii. Platelets count

viii. INR

ix. D-dimers

x. Ferritin

xi. pH

xii. HCO3

xiii. ALT

xiv. AST

xv. CK

xvi. PaO2

*4. Characteristics and cause of AKI*

a. Community acquired/Hospital acquired

b. Days between COVID-19 and diagnosis of AKI

c. Etiology of AKI

i. Fluid depletion/shock

ii. Multiorgan dysfunction syndrome due to sepsis or pro-anti-inflammatory cascade

iii. Rhabdomyolysis

iv. Nephrotoxic drugs / contrast

v. Others id. Diuresis (oliguric / non-oliguric)

d. Serum creatinine peak

e. Kidney replacement therapy (yes/no)

i. Conventional HD

ii. SLED

iii. Continuous KRT

iv. HDF online

v. Hemoadsorption

vi. DP

f. The patient had indication of KRT but did not receive it (yes/no)

g. Recovery of renal function (yes / no)

h. Duration of AKI (if recovered)

*5. Process of care*

a. ICU admission (yes / no)

b. Days in ICU

c. The patient had ICU indication but was not admitted

d. Invasive mechanical ventilation

e. Worst Pa / FiO2

f. Worst PEEP

g. The patient had indication of mechanical ventilation but was not performed

*6. Condition at hospital discharge*

a. Dead

b. Alive discharge from hospital

c. Transferred to another hospital

d. Scr at discharge

e. Hospital length-of stay
