# Supplementary material for: Latin American registry of renal involvement in COVID-19 disease. The relevance of assessing proteinuria throughout the clinical course
Source: PLoS One. 2022 Jan 27;17(1):e0261764. doi: 10.1371/journal.pone.0261764 (PMC8794101; doi:10.1371/journal.pone.0261764)
Supplement: S2 Table — Answers are expressed in percentage. (DOCX) [file pone.0261764.s003.docx]

# **S2 Table. Results of an open survey of Latin-American nephrologist participants and no participants of the Registry coming from 14 countries. Answers are expressed in percentage**

| Gender  Male  Female | 46.4  53.6 |
| --- | --- |
| Position of responders  Head of department  Nephrologist (ward)  Intensivists  Other | 19.3  68.5  2.9  9.3 |
| Did you modify your activity during the pandemic because being a risk group for COVID-19?  Yes  No | 35  65 |
| Did you modify your activity during the pandemic not being risk group for COVID-19 by your own?  Yes  No | 5  95 |
| Did you was regularly provided by PPE in your hospital?  Yes  No  Occasionally | 60.9  34.1  5.0 |
| Did you always had the necessary supplies to perform KRT in patients with any cause AKI?  Yes  No  Occasionally | 53.3  16.0  30.7 |
| Did you assist COVID-19 patients needing KRT but who did not receive it?  Very frequently  Frequently  Occasionally  No | 9.5  8.8  27.0  54.7 |
| Did you assist COVID-19 patients needing intensive care that were not admitted in ICU?  Yes  No | 27.3  72.7 |
| Did you assist COVID-19 patients needing mechanical ventilation but who did not receive it?  Yes  No | 21.6  78.4 |
| In your hospital, regular dialysis schedule was modified in order to increase the renal replacement capacity for patients with any cause AKI?  Yes  No | 38.1  61.9 |
| In your hospital, was PD incorporated or increased for the treatment of patients with any cause? AKI  Yes  No | 10.1  89.9 |
| Has a health care worker of your hospital been infected with COVID-19 because of their practice?  Yes  No | 77.9  22.1 |
| Has a health care worker of your hospital died of COVID-19 infection?  Yes  No | 39.3  60.7 |
