# Supplementary material for: Latin American registry of renal involvement in COVID-19 disease. The relevance of assessing proteinuria throughout the clinical course
Source: PLoS One. 2022 Jan 27;17(1):e0261764. doi: 10.1371/journal.pone.0261764 (PMC8794101; doi:10.1371/journal.pone.0261764)
Supplement: S3 Table — (DOCX) [file pone.0261764.s004.docx]

# **S3 Table. Features of patients with recovered and non-recovered renal function.**

|  | Recovered renal function | Non-recovered renal function | *P* |
| --- | --- | --- | --- |
| Age years, median (IQR) | 61 (50-69) | 65 (56-75) | <0.001 |
| Male sex | 185 (64.2) | 377 (70.9) | 0.031 |
| Comorbidities  Hypertension  Diabetes  Cardiovascular disease  Chronic kidney disease  Obesity  None | 161 (55.7)  102 (35.3)  28 (9.7)  29 (10.0)  86 (29.8)  44 (15.2) | 354 (66.4)  224 (42.0)  99 (18.6)  86 (16.1)  181 (34.0)  56 (10.5) | 0.002  0.035  <0.001  0.010  NS  <0.001 |
| Condition at admission  Mild  Moderate  Severe | 61 (21.2)  130 (45.1)  97 (33.7) | 51 (9.6)  234 (44.0)  247 (46.4) | <0.001  NS  <0.001 |
| Proteinuria at admission | 59 (49.2) | 172 (72.0) | <0.001 |
| *De novo* proteinuria | 15 (31.3) | 20 (55.6) | 0.022 |
| sCr peak (mg/dL) | 2.15 (1.50-4.10) | 4.20 (3.00-5.91) | <0.001 |
| HA-AKI* | 138 (47.8) | 391 (73.5) | <0.001 |
| Etiology of AKI  Volume depletion  SARS-Co-V-2 MODS**  Sepsis MODS | 153 (52.9)  132 (45.7)  62 (21.5) | 153 (28.7)  366 (68.7)  184 (34.5) | <0.001  <0.001  <0.001 |
| Non-oliguric AKI | 202 (70.4) | 281 (53.6) | <0.001 |
| Kidney replacement therapy | 78 (27.1) | 312 (59.6) | <0.001 |
| ICU | 161 (56.9) | 4440 (85.6) | <0.001 |
| Mechanical ventilation | 155 (54.4) | 454 (88.0) | <0.001 |
| Lowest Pa/FiO_2_ (mmHg) | 153 (120-200) | 123 (108-158) | <0.001 |
| Vasopressors | 121 (43.8) | 390 (75.6) | <0.001 |
| No hospital complications | 81 (29.2) | 71 (14.0) | <0.001 |
| Length-of-hospital stay (days) | 16 (10-30) | 121 (7-20) | <0.001 |
| In-hospital mortality | 60 (20.8) | 462 (86.7) | <0.001 |

Variables are presented as number and proportion or median (interquartile range). HA-AKI= hospital-acquired acute kidney injury; MODS= multiorgan dysfunction syndrome; sCr=serum creatinine; ICU=intensive care unit.
