# Supplementary material for: Latin American registry of renal involvement in COVID-19 disease. The relevance of assessing proteinuria throughout the clinical course
Source: PLoS One. 2022 Jan 27;17(1):e0261764. doi: 10.1371/journal.pone.0261764 (PMC8794101; doi:10.1371/journal.pone.0261764)
Supplement: S4 Table — (DOCX) [file pone.0261764.s005.docx]

**S4 Table. Risk factors for mortality in patients assessed for proteinuria at admission. Variables entered in the regression logistic model.**

Age; gender; hypertension; diabetes; cardiovascular disease; obesity; condition at admission; sCr at admission; peak and last values; WBC; setting of AKI; hypovolemia, SARS-CoV-2 MODS, sepsis MODS, and nephrotoxicity as etiological causes, diuresis, KRT, ICU admission, mechanical ventilation, vasopressors, complications; hospital length-of-stay.
