# Supplementary material for: Latin American registry of renal involvement in COVID-19 disease. The relevance of assessing proteinuria throughout the clinical course
Source: PLoS One. 2022 Jan 27;17(1):e0261764. doi: 10.1371/journal.pone.0261764 (PMC8794101; doi:10.1371/journal.pone.0261764)
Supplement: S5 Table — Variables entered in the regression logistic model. (DOCX) [file pone.0261764.s006.docx]

**S5 Table. Risk factors for mortality in patients assessed for proteinuria during hospital stay. Variables entered in the regression logistic model.**

Age; comorbidities; days Covid-19 to AKI; setting of AKI; SARS-CoV-2 and septic MODS as etiological causes of AKI; sCr peak; mechanical ventilation; *de novo* proteinuria; complications.
