# Supplementary material for: Latin American registry of renal involvement in COVID-19 disease. The relevance of assessing proteinuria throughout the clinical course
Source: PLoS One. 2022 Jan 27;17(1):e0261764. doi: 10.1371/journal.pone.0261764 (PMC8794101; doi:10.1371/journal.pone.0261764)
Supplement: S6 Table — (DOCX) [file pone.0261764.s007.docx]

# **S6 Table. Clinical characteristics of critically ill patients**

| Variable | ICU  (622) | No ICU  (221) | *P* |
| --- | --- | --- | --- |
| Obesity n (%) | 227 (35.5) | 40 (18.1) | 0.000 |
| Proteinuira at admission | 195/295 (66.1) | 46/91 (50.5) | 0.023 |
| Hospital-acquired acute kidney injury | 445/620 (71.8) | 90/201 (44.8) | 0.000 |
| Cause of acute kidney injury  SARS-CoV-2 MODS*  Sepsis MODS  Nephrotoxic drugs | 432 (69.5)  229 (36.8)  113 (18.2) | 66 (29.9)  19 (8.6)  65 (29.4) | 0.000  0.000  0.002 |
| Days COVID-hospital admission | 2 (1-5) | 1 (1-3) | 0.000 |
| sCr at admission | 1.10 (0.89-1.61) | 1.50 (1.10-2.67 | 0.000 |
| White blood cells count mm^3^ | 10700 (7800-14700) | 9600 (7295-13397) | 0.028 |
| Ferritine ng/mL | 1291 (750-2000) | 647 (349-1190) | 0.000 |
| pH | 7.39 (7.30-7.42) | 7.41 (7.30-7.45) | 0.004 |
| HCO3 mmol/L | 22 (19-24) | 21 (18-24) | 0.034 |
| Days COVID-AKI onset | 4 (2-9) | 1 (1-3) | 0.000 |
| Last available sCr mg/dL | 3.00 (1.43-4.40) | 1.20 (0.90-2.20) | 0.006 |
| Mortality | 314 (50.6) | 35 (15.8) | 0.000 |

Variables are presented as number and proportion or median (interquartile range). MODS= multiorgan dysfunction syndrome; sCr=serum creatinine.
